# Prognostic effect of high-density lipoprotein cholesterol level in patients with atherosclerotic cardiovascular disease under statin treatment

**DOI:** 10.1038/s41598-020-78828-8

**Published:** 2020-12-14

**Authors:** Yi-Heng Li, Wei-Kung Tseng, Wei-Hsian Yin, Fang-Ju Lin, Yen-Wen Wu, I-Chang Hsieh, Tsung-Hsien Lin, Wayne Huey-Herng Sheu, Hung-I Yeh, Jaw-Wen Chen, Chau-Chung Wu, Taiwanese Secondary Prevention for Patients with AtheRosCLErotic Disease (T-SPARCLE) Registry Investigators

**Affiliations:** 1 Division of Cardiology, Department of Internal Medicine, National Cheng Kung University Hospital, College of Medicine, National Cheng Kung University, Tainan, Taiwan; 2 Department of Medical Imaging and Radiological Sciences, I-Shou University, Kaohsiung, Taiwan; 3 Division of Cardiology, Department of Internal Medicine, E-Da Hospital, Kaohsiung, Taiwan; 4 National Yang Ming University School of Medicine, Taipei, Taiwan; 5 Division of Cardiology, Heart Center, Cheng-Hsin General Hospital, Taipei, Taiwan; 6 Graduate Institute of Clinical Pharmacy and School of Pharmacy, College of Medicine, National Taiwan University, Taipei, Taiwan; 7 Department of Pharmacy, National Taiwan University Hospital, Taipei, Taiwan; 8 Cardiology Division, Cardiovascular Medical Center, Far Eastern Memorial Hospital, New Taipei City, Taiwan; 9 Department of Medical Research and Education, Taipei Veterans General Hospital, Taipei, Taiwan; 10 Cardiology Division, Department of Internal Medicine, National Taiwan University Hospital and National Taiwan University College of Medicine, Taipei, Taiwan; 11 Division of Cardiology, Department of Internal Medicine, Chang Gung Memorial Hospital and Chang Gung University College of Medicine, Taoyuan, Taiwan; 12 Division of Cardiology, Department of Internal Medicine, Kaohsiung Medical University Hospital, Kaohsiung, Taiwan; 13 Faculty of Medicine, College of Medicine, Kaohsiung Medical University, Kaohsiung, Taiwan; 14 Division of Endocrinology and Metabolism and Department of Medical Research, Taichung Veterans General Hospital, Taichung, Taiwan; 15 Division of Cardiology, Department of Internal Medicine, Mackay Memorial Hospital, Mackay Medical College, New Taipei City, Taiwan; 16 Institute of Pharmacology, National Yang Ming University, Taipei, Taiwan; 17 Graduate Institute of Medical Education and Bioethics, College of Medicine, National Taiwan University, Taipei, Taiwan

**Keywords:** Cardiology, Risk factors

## Abstract

In patients with atherosclerotic cardiovascular disease (ASCVD) under statin treatment, the influence of on-treatment level of high-density lipoprotein cholesterol (HDL-C) on cardiovascular (CV) events is controversial. Statin-treated patients were selected from the Taiwanese Secondary Prevention for patients with AtheRosCLErotic disease (T-SPARCLE) Registry, a multicenter, observational study of adult patients with ASCVD in Taiwan. Low HDL-C was defined as < 40 mg/dL for men and < 50 mg/dL for women. The primary outcome was a composite CV events including CV death, myocardial infarction (MI), stroke or cardiac arrest with resuscitation. A total of 3731 patients (mean age 65.6 years, 75.6% men) were included. Patients with on-treatment low HDL-C (44%, mean HDL-C 34.9 ± 6.8 mg/dL) were younger and with more diabetes and higher body weight. The mean follow-up time was 2.7 years. We used restricted cubic spline curves to examine the potential non-linear association between HDL-C and adverse outcomes. Decreased HDL-C levels were associated with a significantly increased risk of CV events in women (< 49 mg/dL in women) but not in men (< 42 mg/dL in men). However, the protective effect of elevated HDL-C levels was more prominent in men than in women. In ASCVD patients with statin therapy, low on-treatment HDL-C was common in Taiwan and associated with an increased risk of CV events in women. Higher HDL-C levels provided more protective effect in men than in women.

## Introduction

Multiple lines of evidence have established the role of low-density lipoprotein cholesterol (LDL-C) in the development and progression of atherosclerotic cardiovascular disease (ASCVD)^1^. Lower levels of LDL-C have been shown to reduce cardiovascular (CV) events in multiple clinical trials and human mendelian genetic studies^2,3^. However, a significant residual CV risk remains in patients under intensive statin therapy with controlled LDL-C levels^4,5^. Previous epidemiological studies demonstrated that a low level of high-density lipoprotein cholesterol (HDL-C) is a risk factor of ASCVD^6,7^. In a meta-analysis of 20 randomized clinical trials of statins, a significant inverse association between on-treatment HDL-C levels and the CV risk was observed in the placebo group as well as in the statin groups^8^. But almost all subsequent clinical trials with pharmacological therapies to raise HDL-C in statin-treated patients failed to improve clinical outcomes despite substantial increases in HDL-C levels^9^. Human mendelian genetic study also found genetic polymorphisms that raise plasma HDL-C levels were not associated with a lower risk of acute myocardial infarction (MI)^10^. Low HDL-C is a common lipid phenotype in Asia. Population studies in the Asia–Pacific region showed that the prevalence of low HDL-C was 33.1% in Asians which was significantly higher than that in non-Asians^11^. However, few studies have evaluated the clinical importance of HDL-C levels in Asian patients treated with statins. The Taiwanese Secondary Prevention for patients with AtheRosCLErotic disease (T-SPARCLE) registry is a large-scale observational study to recruit and follow up patients with ASCVD who have been receiving secondary prevention therapies in Taiwan^12^. Using the T-SPARCLE registry data, we evaluated whether on-treatment HDL-C levels carry any prognostic significance in patients with ASCVD under statin treatment. The specific aims of this study were to (1) evaluate the prevalence and determining factors of low HDL-C in patients with ASCVD treated with statins and (2) assess whether the low on-treatment HDL-C is an important predictive factor of adverse clinical outcomes in these patients.

## Methods

### Patient population

We used the data of patients who were under statin treatment at enrollment in the T-SPARCLE registry to perform the study. The T-SPARCLE registry is a prospective, multicenter, observational study that included adult ASCVD patients from 14 major hospitals across Taiwan. The study protocol has been described previously^12^. In this study, the inclusion criteria were patients who were (1) age > 18 years; (2) with stable symptomatic ASCVD, including coronary artery disease, cerebrovascular disease or peripheral artery disease; (3) taking a statin; (4) willing to follow a National Cholesterol Education Program (NCEP) therapeutic lifestyle change or similar cholesterol-lowering diet; and (5) willing to provide informed consent. The exclusion criteria were patients with (1) other serious heart disease; (2) ≥ New York Heart Association functional class III heart failure; (3) life-threatening malignancy; (4) treatment with immunosuppressive agents; (5) other atherosclerotic vascular diseases with unknown disease type; (6) two or more statins treatment at enrollment; (7) chronic dialysis or (8) any condition or situation which, in the opinion of the investigators, might be not suitable for this registry study. The patients’ demographic data, major vascular risk factors, previous disease history, and medications were collected according to a predetermined protocol. The body mass index (BMI) was calculated as the body weight divided by the square of the body height (kg/m^2^). Hypertension, diabetes, heart failure, and ASCVD were diagnosed following conventional definitions and confirmed by the physicians that recruited the study participants. Laboratory test results, including creatinine levels, total cholesterol (TC), triglyceride (TG), LDL-C and HDL-C were obtained after enrollment. Low HDL-C was defined as < 40 mg/dL for male and < 50 mg/dL for female. Serum creatinine was used to calculate estimated glomerular filtration rate (eGFR) by using the Modification of Diet in Renal Disease equation. Chronic kidney disease (CKD) was defined as patients with eGFR < 60 mL/min/1.73m^2^. The definition of statin intensity was based on the recommendation of the 2013 American College of Cardiology/American Heart Association cholesterol guideline^13^. Written informed consent was obtained from each patient included in the study. The study protocol conforms to the ethical guidelines of the 1975 Declaration of Helsinki and was approved by the Taiwan Joint Institutional Review Board for each participating hospital (JIRB number 09-S-015).

### Follow-up

The eligible patients who fulfilled the enrollment criteria were followed up in the original hospitals that recruited the patients. The follow-up information of the patients was collected every year after enrollment. In this study, the primary outcome was a composite endpoint of CV death, nonfatal stroke, nonfatal MI, or cardiac arrest with resuscitation. The follow-up time in the analysis was from the enrollment to the occurrence of first primary outcome event or till the last follow-up visit if there was no primary outcome occurred.

### Statistical analysis

Continuous variables were presented as means ± standard deviations (SDs) and categorical variables were presented as numbers and percentages. The intergroup comparisons were made by using chi-square test with or without Yates’ correction for categorical variables and unpaired Student’s *t* test or Mann–Whitney rank sum test for continuous variables. A 2-tailed *p* value < 0.05 was considered significant. The influence of HDL-C on primary outcome was analyzed by using Cox proportional hazards models. The HDL-C levels were analyzed in the models either as a categorical variable or as a continuous variable. Multivariate Cox proportional hazard analysis was used to identify independent predictors of the primary outcome while adjusting multiple clinical factors, including age, sex, BMI, eGFR, vascular risk factors, smoking status, MI, ischemic stroke/transient ischemic attack, coronary/peripheral intervention and concomitant medications. When HDL-C was treated as a continuous variable, restricted cubic splines was used to produce a curve of hazard ratio (HR) between HDL-C and risk of primary outcome. Three knots at 0.25, 0.50, and 0.75 were used in the splines and the HR between two points of a continuous variable was estimated. We used SAS statistical package (version 9.4 for Windows; SAS Institute, Cary, NC, USA) for all analyses.

## Results

**Figure 1:**
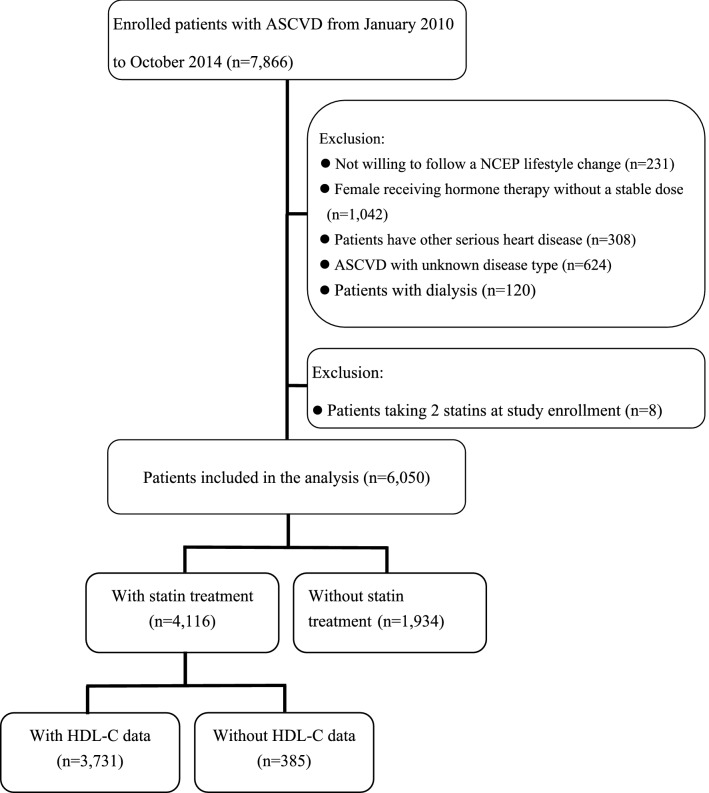
Flow chart for the inclusion and exclusion of study patients. ASCVD, atherosclerotic cardiovascular disease; HDL-C, high-density lipoprotein cholesterol; NCEP, National Cholesterol Education Program.

**Figure 2:**
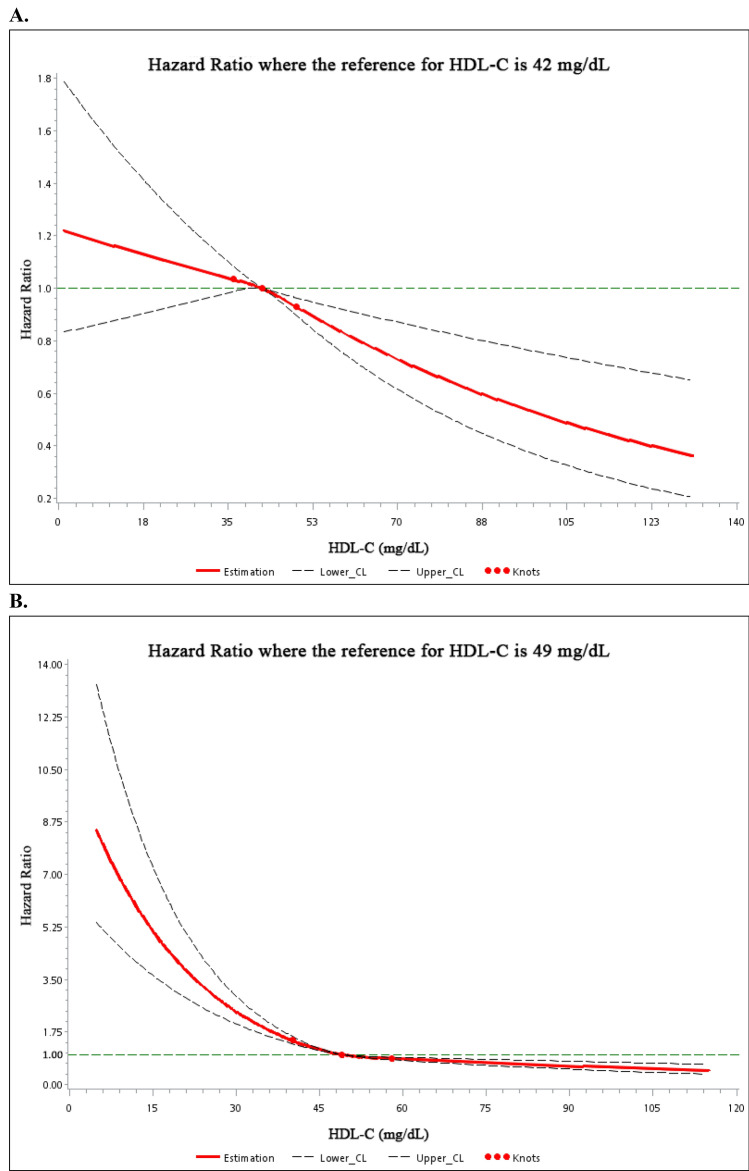
Hazard ratio of HDL-C for risk of primary outcome using restricted cubic spline Cox model analysis in men (**A**) and women (**B**). CL, confidence limit; HDL-C, high-density lipoprotein cholesterol.

Overall, 7866 ASCVD patients were screened from January 2010 to October 2014. After exclusion, 6050 patients were enrolled and 3731 of them (mean age 65.6 ± 11.3 years, 75.6% men) who were taking a statin at enrollment formed the basis of this analysis (Fig. 1). Among them, 1629 (43.7%) patients had low HDL-C under statin treatment. Table 1 shows the clinical characteristics of patients with and without low HDL-C under statin treatment. Patients with low HDL-C were younger and had more female and higher BMI. More of them had a history of diabetes mellitus, coronary or peripheral intervention, coronary artery disease and MI. The TG level was also higher in patients with low HDL-C. Multivariate logistic analysis found that younger age, female, diabetes, smoking, use of beta-blocker, increased BMI and CKD with eGFR ≤ 30 ml/min/1.73m^2^ were the independent predicting factors of low HDL-C level (Table 2). Statin intensity was not a predicting factor of low HDL-C. The mean total follow-up time was 2.7 year. During this period, 110 (3.0%) patients developed the primary outcome events, including 22 patients with CV death, 37 with nonfatal stroke, 44 with nonfatal MI and 7 with cardiac arrest with resuscitation. Compared with those without events, the patients who developed outcome events were older and had lower BMI, more heart failure, diabetes and CKD (Table 3). The HDL-C levels were similar between patients with and without outcome events (44.0 ± 13.1 vs. 44.9 ± 12.9 mg/dL, *p* = 0.47). In the multivariate Cox proportional hazard analysis after adjusting for all clinical factors, we found age, smoking, without beta-blocker use, heart failure and CKD with eGFR ≤ 30 ml/min/1.73m^2^ were independent predictors of the primary outcome. Low HDL-C was not significantly associated with an increased risk of the primary outcome (normal vs. low HDL-C, HR, 0.83, 95% confidence interval [CI], 0.56–1.23) (Table 4). Statin intensity had no association with clinical outcome. When the level of HDL-C was analyzed as a continuous variable by using restricted cubic splines, a significantly negative association of HDL-C with the risk of primary outcome was observed in women with low HDL-C levels (HDL-C < 49 mg/dL), but not in men (HDL-C < 42 mg/dL) (Fig. 2). On the contrary, elevated HDL-C levels were continuously associated with a decreased risk of outcome events in men (HDL-C > 42 mg/dL) and the protective effect of elevated HDL-C was much more evident in men than in women (Fig. 2).

**Table 1:** Baseline characteristics of patients with normal or low HDL-C.

Variables, n (%)	Normal HDL-C (n = 2102)	Low HDL-C (n = 1629)	*p* value
Age (yrs)	66.2 ± 11.1	64.7 ± 11.5	< 0.0001
Gender (Male )	1659 (78.9)	1160 (71.2)	< 0.0001
BMI (kg/m2)	25.9 ± 3.6	27.0 ± 3.9	< 0.0001
Cigarette smoking history	963 (45.9)	773 (47.5)	0.33
With anti-platelets therapy	1909 (90.8)	1487 (91.3)	0.66
With ARB/ ACEI	1228 (58.4)	974 (59.8)	0.42
With beta-blocker	1167 (55.5)	1051 (64.5)	< 0.0001
History of hypertension	1457 (69.7)	1178 (72.3)	0.06
History of heart failure	228 (10.9)	197 (12.1)	0.26
History of diabetes mellitus	633 (34.3)	684 (46.4)	< 0.0001
Previous coronary/peripheral intervention	1234 (58.7)	1034 (63.5)	0.0034
Coronary artery disease	1955 (93.0)	1555(95.5)	0.0021
Myocardial infarction	1679 (79.9)	1364 (83.7)	0.003
Ischemic stroke/TIA	240 (11.4)	159 (9.8)	0.17
Chronic kidney disease	505 (25.9)	402 (26.9)	0.52
**Lipid profile (mg/dL)**			
Total cholesterol	174.2 ± 39.7	159.2 ± 36.5	< 0.0001
Triglyceride	118.7 ± 79.9	162 ± 93.0	< 0.0001
LDL-C	98.2 ± 33.96	92.8 ± 34.2	< 0.0001
HDL-C	52.6 ± 11.2	34.9 ± 6.8	< 0.0001

*ACEI* angiotensin converting enzyme inhibitor, *ARB* angiotensin receptor blocker, *BMI* body mass index, *HDL-C* high-density lipoprotein cholesterol, *LDL-C* low-density lipoprotein cholesterol, *TIA* transient ischemic attack.

**Table 2:** Multivariate analysis for predicting factors of low HDL-C.

Parameter	Hazard ratio	95% CI	*p* value
Age	0.99	0.98–1.00	< 0.01
Male (vs. female)	0.52	0.44–0.62	< 0.0001
History hypertension	1.05	0.90–1.23	0.52
History of diabetes mellitus	1.54	1.33–1.77	< 0.0001
History of heart failure	1.08	0.88–1.34	0.46
History of myocardial infarction	1.33	1.10–1.60	< 0.01
Previous coronary/peripheral intervention	1.12	0.97–1.30	0.13
History of ischemic stroke/TIA	0.97	0.77–1.22	0.78
Cigarette smoking history	1.23	1.05–1.44	0.01
**Statin intensity (vs. moderate intensity)**			
Low intensity	0.94	0.78–1.14	0.55
High intensity	0.87	0.64–1.18	0.37
Anti-platelets therapy	0.98	0.77–1.26	0.90
ARB/ACEI	0.97	0.84–1.11	0.62
Beta-blocker	1.35	1.18–1.56	< 0.0001
**BMI (vs. BMI ≥ 27.5 )**			
BMI < 23	0.49	0.40–0.61	< 0.0001
23 ≤ BMI < 27.5	0.74	0.64–0.87	0.0001
**eGFR (vs. eGFR > 60 ml/min/1.73m**^2^**)**			
30 < eGFR ≤ 60	1.01	0.85–1.20	0.92
eGFR ≤ 30	1.58	1.01–2.50	0.05

*Abbreviations are the same as the Table 1. eGFR, estimated glomerular filtration rate.

**Table 3:** Baseline characteristics in patients with and without primary outcome events.

Variables, n (%)	With outcome events (n = 110)	Without outcome events (n = 3621)	*p* value
Age (yrs)	69.7 ± 12.4	65.4 ± 11.2	0.0001
Gender (Male )	78 (70.9)	2741 (75.7)	0.30
BMI (kg/m2)	25.5 ± 3.6	26.4 ± 3.7	0.0193
Cigarette smoking history	60 (54.6)	1676 (46.3)	0.11
With anti-platelets therapy	101 (91.8)	3295 (91.0)	0.90
With ARB/ ACEI	72 (65.5)	2130 (58.8)	0.19
With beta-blocker	47 (42.7)	2171 (60.0)	< 0.0005
History of hypertension	80 (72.7)	2555 (70.6)	0.71
History of heart failure	25 (22.7)	400 (11.1)	< 0.0005
History of diabetes mellitus	52 (49.5)	1265 (39.3)	0.0453
Previous coronary/peripheral intervention	65 (59.1)	2203 (60.8)	0.79
Coronary artery disease	103 (93.6)	3407 (94.1)	0.84
Myocardial infarction	96 (87.3)	2947 (81.4)	0.15
Ischemic stroke/TIA	19 (17.3)	380 (10.5)	0.0349
Chronic kidney disease	49 (47.1)	858 (25.7)	< 0.0001
**Lipid profile (mg/dL)**			
Total cholesterol	173.3 ± 38.8	167.5 ± 42.0	0.12
Triglyceride	148.9 ± 95.4	137.3 ± 88.3	0.17
LDL-C	99.1 ± 36.9	95.7 ± 34.1	0.31
HDL-C	44.0 ± 13.1	44.9 ± 12.9	0.47
**Primary outcome events**			
Cardiovascular death	22 (20.0)	–	
Nonfatal stroke	37 (33.6)	–	
Nonfatal myocardial infarction	44 (40.0)	–	
Cardiac arrest with resuscitation	7 (6.4)	–	

*Abbreviations are the same as the Table 1.

**Table 4:** Multivariate analyses for predicting factors of primary outcome events.

Parameter	Hazard ratio	95% CI	*p* value
Age	1.02	1.00–1.04	0.03
Male (vs. female)	0.71	0.42–1.20	0.20
History hypertension	0.97	0.62–1.52	0.91
History of diabetes mellitus	1.36	0.92–2.01	0.13
History of heart failure	2.08	1.30–3.31	< 0.01
History of myocardial infarction	1.73	0.94–3.22	0.08
Previous coronary/peripheral intervention	1.16	0.76–1.75	0.49
History of ischemic stroke/TIA	1.54	0.90–2.62	0.12
Cigarette smoking history	1.85	1.15–2.97	0.01
**Statin intensity (vs. Moderate intensity)**			
Low intensity	0.91	0.53–1.56	0.72
High intensity	0.55	0.17–1.73	0.30
Anti-platelets therapy	1.19	0.59–2.39	0.63
ARB/ACEI	1.25	0.83–1.88	0.28
Beta-blocker	0.56	0.37–0.82	< 0.01
**BMI (vs. BMI ≥ 27.5)**			
BMI < 23	1.45	0.84–2.49	0.18
23 ≤ BMI < 27.5	0.88	0.55–1.41	0.61
**eGFR (vs. eGFR > 60 ml/min/1.73m**^2^**)**			
30 < eGFR ≤ 60	1.44	0.92–2.25	0.11
eGFR ≤ 30	4.33	2.20–8.50	< 0.0001
Normal HDL-C level (vs. Low HDL-C)	0.83	0.56–1.23	0.35

*Abbreviations are the same as the Table 1. eGFR, estimated glomerular filtration rate.

## Discussion

The major findings of this study were: (1) low HDL-C was a common lipid phenotype in patients with ASCVD treated with statins, (2) statin intensity was not associated with low HDL-C, (3) low HDL-C was associated with an increased risk of adverse clinical outcomes in female patients with ASCVD under statin treatment, and (4) a more protective effect of increased HDL-C was observed in men than in women.

In this Asian cohort, we found low HDL-C was a common lipid phenotype (43.7%) in patients under statin treatment. In the Treating to New Targets (TNT) study, 9770 patients with stable coronary artery disease were randomized to receive 10 mg or 80 mg/day atorvastatin therapy. At 3 months after treatment, 57.8% patients had HDL-C < 48 mg/dL^14^. In the 3574 patients with acute MI in the Korea Acute Myocardial Infarction Registry, more than 75% of these patients received statin treatment and 45.5% patients had HDL-C < 40 mg/dL at 12-month follow-up after discharge^15^. Although HDL-C raising ability was different among statins^16,17^, we found there was no relation between the presence of low HDL-C level and statin intensity. In recent decades, there were great changes of lipid phenotype between Asian and Western countries. Population-based studies demonstrated that there was an increased TC in Asian countries, but declined in most Western countries^18^. The increase of TC in Japan and South Korea was mainly due to an increase of HDL-C. But in China, a rise in non-HDL-C was the reason for increased TC. In most Western countries, there was mainly a decrease of non-HDL-C and an increase of HDL-C^18^. The differences in genetic background, economic development, dietary habit changes, and prescription rate of lipid lowering drugs among the different regions cause the variations of lipid phenotype. All these factors also cause complexity of the relationship between HDL-C and CV outcome.

The prognostic role of HDL-C in statin-treated patients is still controversial. The post-hoc analysis of the TNT study showed that an increase of 1 mg/mL in HDL-C reduced the risk of major CV events by 1.1% (*p* = 0.003). However, there was no significant difference in CV risk between the highest and the lowest quintile of HDL-C in high-intensity statin treated patients^14^. By analyzing HDL-C in categories, both the Pravastatin or Atorvastatin Evaluation and Infection Therapy–Thrombolysis in Myocardial Infarction 22 (PROVE IT–TIMI 22) trial and Justification for the Use of Statins in Prevention: an Intervention Trial Evaluating Rosuvastatin (JUPITER) trials found the on-treatment HDL-C level had no significant predictive value for subsequent CV risk in patients with statin therapy^19,20^. In our study, by treating HDL-C as a continuous variable, restricted cubic spline curves showed that decreased HDL-C levels were associated with increased risk of primary outcome in female patients. The protective effects of elevated HDL-C was more prominent in men than in women. Gender has great influence on the relation of HDL-C to the risk of ASCVD. In the Los Angeles Atherosclerosis Study (LAAS), after adjusting potential confounders in the multivariate models, the progression of carotid intima-media thickness was inversely associated with serum levels of HDL-C in men. For women, the protective effect of HDL-C level diminished in the age of menopause^21^. In the 1380 females from the Multi-Ethnic Study of Atherosclerosis (MESA) study, the higher HDL-C was not even protective but associated with a greater risk for predicting the presence of carotid plaque in postmenopausal women^22^. One of the explanations for the different HDL-C effect of female is related to estrogen reduction in menopause. Gradual decrease of the antioxidant effect of estrogen over the menopausal transition increases formation of reactive oxygen species and changes the HDL-C composition resulting in less anti-inflammatory and antioxidant properties of HDL-C^23^. Another problem of the current measurement of HDL-C, the cholesterol carried by HDL particles, may not reflect the true effect of HDL on CV risk. Other HDL-related biomarkers, such as HDL function or HDL particle number, may carry more important clinical significance than HDL-C levels. In the JUPITER trial, the HDL particle number and HDL cholesterol efflux capacity had a statistically significant and stronger negative association with CV disease than HDL-C levels among rosuvastatin-treated individuals^24^.

The major limitation of our study is its observational study design. The confounding factors that could not be identified or measured potentially may bias the clinical outcomes. For example, we did not know the severity of coronary stenosis in patients with coronary artery disease. The data of left ventricular ejection fraction were also unknown. These unmeasured confounding factors could influence the clinical outcomes and bias the study results. Second, information bias including the drug compliance and clinical endpoint identification during the clinical follow up was another study limitation. Third, the follow-up time was still short. The long term effect of low HDL-C in these patients was unknown. Finally, the status of menopause in female patients was not known in our study. We found gender played an important role and low HDL-C was associated with increased risk of adverse clinical outcomes only in female patients. However, the influence of menopause could not be analyzed in our study.

In conclusion, this observational study indicates that low on-treatment HDL-C is a common lipid phenotype in statin-treated patients in Taiwan. It is associated with increased risk of adverse clinical outcomes in female patients with ASCVD. The protective effect of increased HDL-C is more evident in men than in women.
